# Editorial: New perspectives and innovative techniques in contemporary spine surgery

**DOI:** 10.3389/fsurg.2023.1220181

**Published:** 2023-06-12

**Authors:** Luca Ambrosio, Gianluca Vadalà, Fabrizio Russo, Daisuke Sakai, Vincenzo Denaro

**Affiliations:** ^1^ Operative Unit of Orthopaedic and Trauma Surgery, Fondazione Policlinico Universitario Campus Bio-Medico, Rome, Italy; ^2^ Research Unit of Orthopaedic and Trauma Surgery, Department of Medicine and Surgery, Università Campus Bio-Medico di Roma, Rome, Italy; ^3^ Department of Orthopedic Surgery, Surgical Science, School of Medicine, Tokai University, Isehara, Japan

**Keywords:** spine, minimally invasive spine surgery, navigation, artificial intelligence, osteobiologic

**Editorial on the Research Topic** New Perspective and Innovative Techniques in Contemporary Spine Surgery

Spine surgery is a multidisciplinary field in which the implementation of novel technologies and techniques has fostered significant advancements in the last decade. The utilization of robotics and navigation systems has made it possible to perform accurate planning and minimize intraoperative complications such as screw malpositioning and increased blood loss (1). Furthermore, the increasing use of microscopy and endoscopy, as well as the integration with both preoperative and intraoperative advanced imaging, is continuously contributing to the development of minimally invasive spine surgery (MISS) techniques (2). Among additional breakthroughs, the use of innovative biofabrication technologies is continuously enhancing the surgeon's armamentarium with different biomaterials and osteobiologics based on diverse clinical needs (3). On the other hand, the application of artificial intelligence (AI) and machine learning (ML) is being extensively employed to develop interactive systems able to support clinical decisions and optimize postoperative outcomes (4–6). Nonetheless, the COVID-19 pandemic has posed unique challenges to the spine community, which have reshaped our practice in several different ways (7).

In this Research Topic, several authors have significantly contributed to providing innovative insights and highlighted the potential of groundbreaking technologies that will likely further advance the field in the next future. Bacco et al. systematically reviewed the available evidence on the application of a novel AI tool, namely, natural language processing (NLP), in spine research. An et al. conducted a retrospective analysis of patients affected by gluteal pain due to lumbar disc herniation (LDH) and treated with percutaneous endoscopic transforaminal discectomy or open discectomy, showing that the former was equally effective while reducing operation time, blood loss, hospital stay, and financial burden. Using a similar study design, Wang et al. evaluated the efficacy of an annulus fibrosus suture device used during endoscopic lumbar discectomy, successfully demonstrating a reduction in the risk of LDH recurrence and no additional complications compared with patients receiving endoscopic discectomy alone. In a single-arm retrospective study, Wang et al. showed that unilateral biportal endoscopic transforaminal lumbar interbody fusion (TLIF) was significantly effective in reducing pain and disability in patients with lumbar spine stenosis, offering intriguing advantages over traditional techniques. In their study, Wu et al. compared the clinical outcomes of patients affected by degenerative spondylolisthesis and treated with oblique lumbar interbody fusion (OLIF) and TLIF, showing lower blood loss, reduced cage subsidence, and increased disc height in the former group. Manufacturing and production of novel osteobiologics to promote bone fusion are crucial for enhancing clinical outcomes following spine surgery. In their study, Aurouer et al. reported successful fusion in >90% of patients undergoing anterior cervical discectomy and fusion and anterior lumbar interbody fusion augmented with supercritical CO_2_-processed bone allografts, in the absence of adverse events.

This Research Topic also included reports of unusual cases of spinal disorders and preliminary reports of novel surgical techniques. Ding et al. reported a rare case of spinal involvement in a patient affected by alkaptonuria and severe thoracolumbar stenosis, which was effectively treated with surgical decompression and instrumentation. Meng et al. illustrated a case of severe post-traumatic kyphosis due to an AO type B2.3 T12 fracture successfully treated with posterior hemivertebra resection and segmental fixation. Conversely, Rui et al. compared traditional open pedicle screw fixation for single-level thoracolumbar fractures with percutaneous screw placement augmented with allogeneic bone graft following vertebral body distraction. Intriguingly, this novel technique resulted in lower blood loss, decreased operative time, a reduction of costs, length of stay, and incision length, as well as a higher vertebral height. In their study, Huang et al. described an innovative approach to treat multilevel cervical spondylotic myelopathy based on a modification of the open-door laminoplasty technique, which was performed on alternate sides of the laminae instead of unilaterally. Zou et al. reported the preliminary results of the application of a novel reduction plate specifically manufactured for unstable atlas fractures to be treated via an anterior transoral approach. This surgical approach was also employed by the same authors to develop a novel surgical technique to treat irreducible atlantoaxial dislocations in pediatric patients through intra-articular cage distraction and fusion with a C-JAWS stapler. On the other hand, Miao et al. compared the surgical outcomes of partial C2 laminectomy vs. C2 dome-like laminectomy in patients affected by ossification of the posterior longitudinal ligament, showing that the latter was able to reduce the incidence of neck pain, although the former achieved a wider decompression. In their case series, Xia et al. illustrated a novel technique to treat congenital scoliosis in children aged less than 4 years by means of hemivertebra resection and subsequent prolonged bracing. Although generally feasible and associated with satisfactory outcomes, the authors acknowledged the reduced capacity of this approach to correct thoracolumbar sagittal deformities.

Nonetheless, this Research Topic also included interesting reports on relevant themes in the field of spine surgery. In their study, Lu et al. proposed a modified version of the Thoracolumbar Injury Classification and Severity Score (TLICS). More specifically, the authors suggested implementing an additional subcategory describing the intervertebral disc injury status to underline the importance of the disc complex in vertebral stability. Wang et al. performed a retrospective multicenter review of patients affected by traumatic spinal cord injury in Northwest China. The authors reported an increasing trend of cases in the last few years, followed by a slight reduction due to the COVID-19 pandemic, which provided interesting insights on future strategies to reduce the impact of such a devastating event.
